# Light from a firefly at temperatures considerably higher and lower than normal

**DOI:** 10.1038/s41598-021-91839-3

**Published:** 2021-06-14

**Authors:** Mana Mohan Rabha, Upamanyu Sharma, Anurup Gohain Barua

**Affiliations:** 1 Department of Physics, Pandit Deendayal Upadhyaya Adarsha Mahavidyalaya, Behali, 784184 India; 2 Department of Physics, Gauhati University, Guwahati, 781014 India

**Keywords:** Biological fluorescence, Biological physics

## Abstract

Bioluminescence emissions from a few species of fireflies have been studied at different temperatures. Variations in the flash-duration have been observed and interesting conclusions drawn in those studies. Here we investigate steady-state and pulsed emissions from male specimens of the Indian species *Sclerotia substriata* at temperatures considerably higher and lower than the ones at which they normally flash. When the temperature is raised to 34 °C, the peak wavelength gets red-shifted and the emitted pulses become the narrowest which broaden considerably thereafter for small increases in temperature; this probably indicates denaturation of the enzyme luciferase catalyzing the light-producing reaction. When the temperature is decreased to the region of 10.5–9 °C, the peak gets blue-shifted and the flash-duration increased abnormally with large fluctuation; this possibly implies cold denaturation of the luciferase. We conclude that the first or hot effect is very likely to be the reason of the species being dark-active on hot days, and the second or cold one is the probable reason for its disappearance at the onset of the winter. Our study makes the inference that these two happenings determine the temperature-tolerance, which plays a major role in the selection of the habitat for the firefly.

## Introduction

The light of the firefly is the outcome of a very efficient reaction, called chemiluminescent reaction. It is well-known that oxygen is the biochemical trigger which excites the substrate luciferin, and produces the photo-emitter molecule oxyluciferin in presence of ATP and Mg^2^^+^, the reaction being catalyzed by the enzyme luciferase. In the normal flashing state of a live firefly, visible light is produced as the excited state oxyluciferin decays to the ground state via a pathway followed by molecules indicating phosphorescence^1^. It has been shown that the pulses produced by the firefly are manifestations of an oscillating chemical reaction^2^. Very recently, assuming the firefly lighting cycle to be a nonlinear oscillator with a robust periodic cycle, a low dimensional nonlinear mathematical model based on the basic lighting mechanism of a firefly has been proposed^3^.

A few studies have been carried out on the effect of temperature on in vivo emissions of fireflies. It has been observed that flash periods of four *Luciola* species of fireflies of Melanesia decrease with an increase in temperature^4^. A significant negative correlation between the ambient temperature and inter flash interval has been observed in specimens of *Luciola cruciata* at five different sites in central Japan^5^. For the Indian species *Luciola praeusta* at 20–40 °C, the pulse duration has been found to decrease approximately linearly with temperature for male specimens^6^ and exponentially with temperature for female specimens^7^. These imply that the speed of the light-producing reaction increases approximately linearly for males and exponentially for females of this species in this range of temperature. At the temperature of approximately 42 °C, the duration of a flash from this species becomes the minimum and thereafter increases considerably with a slight increase in the temperature, implying that denaturation of the enzyme occurs at this temperature optimum. The peak wavelength also shows a red shift of about 5 nm at this temperature. On the other hand, at temperatures lower than approximately 21 °C, pulse-durations show large increase in a non-linear manner with lowering of the temperature^8^—almost like the ones observed with fireflies positioned under a strong static magnetic field^9^. Below 17 °C for females and 15 °C for males of this species, and from below the normal flashing temperature of about 28 °C for females and below about 22 °C for males of another Indian species *Asymmetricata circumdata*, peaks in flashes get split into three, manifesting the three luminescent forms of the emitter oxyluciferin, with lifetimes of the order of milliseconds^1^.

**Figure 1:**
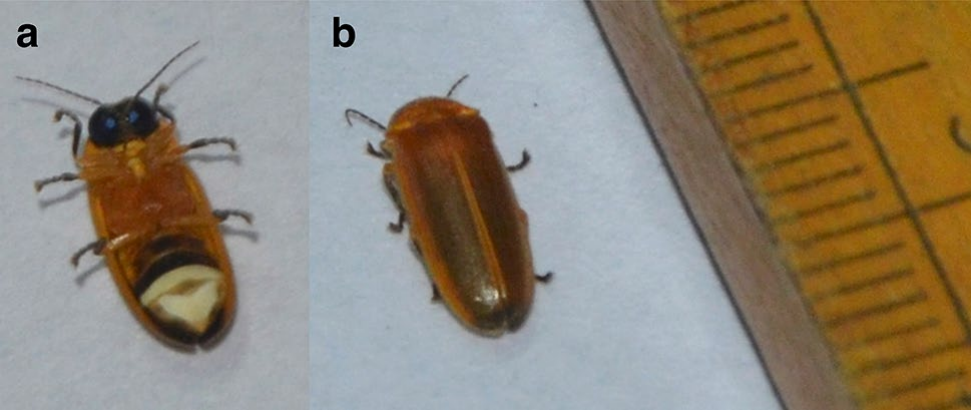
A specimen of male adult firefly *S. substriata*. (**a**) Ventral side. (**b**) Dorsal side. Average lengths and weights of the collected specimens are 9 mm and 10 mg, respectively. In the campus of Gauhati University, this species is mostly found flashing from trees on the banks of two large ponds lying side by side.

*Sclerotia substriata* is the fourth species of firefly found in India after the summer species *L. praeusta* and *A. circumdata*, and the winter one *Diaphanes* sp. This species has been identified by Dr. Lezley Ballantyne of Charles Sturt University, Australia. A male specimen of this species is shown in Fig. 1. It is found near the banks of two large ponds, separated by a lane, in the campus of Gauhati University in very small numbers from April to September, and in even smaller numbers in March and October. The geographic location of the place is 26.1543° N and 91.6632° E. A survey of literature indicates that no study on the characteristics of light from *S. substriata* has been carried out till now. In this article, we present emission spectra and pulses of this species at temperatures substantially higher and lower than its normal flashing ones.

## Results and discussion

### Steady-state measurements at different temperatures

**Figure 2:**
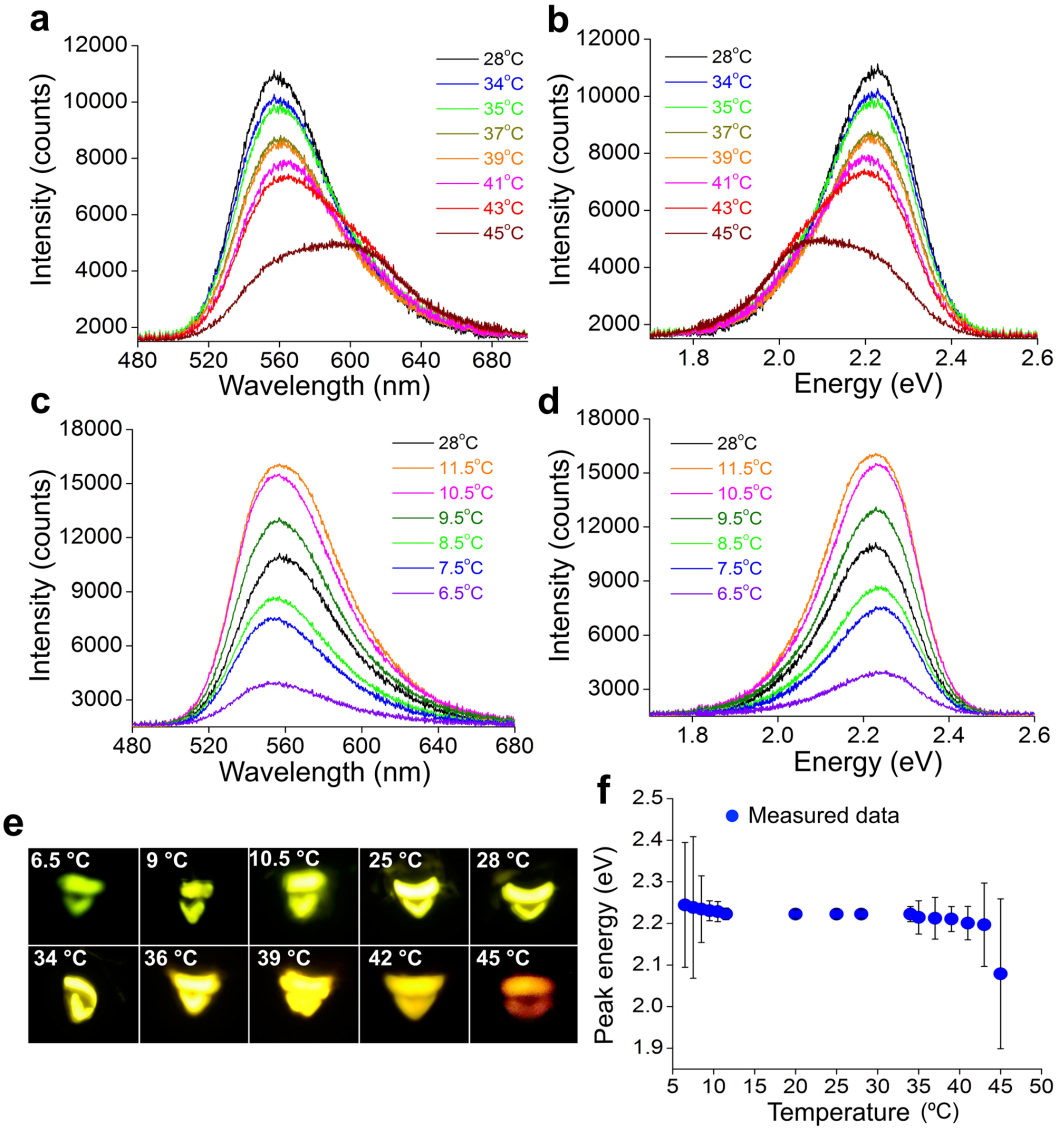
Steady-state emissions from the firefly *Sclerotia substriata*. (**a**,**c**) Bioluminescence spectra in the wavelength scale. At the normal laboratory temperature of 28 ºC, the peak of the spectrum appears at 558 nm with a FWHM value of 61 nm. Values of the peak and the FWHM remain the same up to 34 ºC. As the temperature is gradually increased further, the peak of the spectrum begins to shift towards red and the FWHM begins to get broadened with its lower and upper points also showing red shifts. At 45 ºC, the peak shifts to a maximum value of approximately 598 nm, and the FWHM extends to a maximum value of approximately 96 nm. At low temperatures, values of the peak and FWHM remain the same up to approximately 10.5 °C. Thereafter, the peak shifts towards blue and the FWHM gets narrower. At 6.5 °C, the peak shifts to 553 nm and the FWHM gets narrowed to the value of 57 nm. (**b**,**d**) Bioluminescence spectra in the energy scale. The bioluminescence peak energy, which remains the same up to 34 ºC, decreases slowly up to 41 ºC and thereafter rapidly up to 45 °C. (**e**) Photographs of the firefly light emitting organ at various temperatures. There is no change in the color of the light organ at 25, 28, and 34 ºC, which indicates the same values of peak and FWHM. The color changes to yellow at 36 ºC, to yellow-orange at 39 and 42 ºC, and to red at 45 ºC. On the other hand, the lantern shows tinges of green at 10.5 and 9 °C; the color becomes predominantly green at 6.5 ºC. This figure is a visual indication of shifts in the wavelength peak. Changes in the color from yellowish-green to red and to green make it clear that the peak gets shifted at both high and low temperatures. (**f**) Measured peak energy data (mean ± SD) at various temperatures in the range of 6.5–45 ºC. Standard deviations, that is, fluctuations, are clearly much more at both low and high temperatures compared to those at normal temperatures.

Steady-state emission spectra recorded at high temperatures of 34, 35, 37, 39, 41, 43 and 45 ºC along with the one at the normal laboratory temperature of 28 ºC are shown in the wavelength scale in Fig. 2a, and in the energy scale (eV) in Fig. 2b. Emission spectra at low temperatures of 11.5, 10.5, 9.5, 8.5, 7.5 and 6.5 ºC with the normal one at 28 ºC are presented in the wavelength scale in Fig. 2c, and in the energy scale in Fig. 2d. A typical spectrum in the normal range of temperature for this species is asymmetric in nature. The wavelength peak appears at 558 nm, and the full width at half maximum (FWHM) is measured as 61 nm, spreading from 532.5 to 593.5 nm, at the laboratory temperature of 28 ºC (Supplementary Table 1). It has been hypothesized that different species of fireflies emit light at slightly different wavelength peaks because of slight differences in their enzyme structures^10^.

Changes in temperature from 20 up to 34 ºC do not change the values of the emission peak (558 nm) and FWHM (61 nm). From 34 ºC onwards, the peak begins to shift towards red; the lower and upper positions of the FWHM begin red-shifting as well, and the spread becomes broad. At 45 °C, the maximum employed temperature that only a few of the specimens could withstand, the maximum peak shift of 40 nm with the maximum FWHM spread of 96 nm, ranging from 539 to 635 nm, are observed (Supplementary Table 1). At this temperature, the light organs of the surviving specimens exhibit continuous glows for a couple of minutes rather than on–off emissions, that is, flashes. We may consider this temperature as the maximum tolerable one for this firefly. Variations in emission wavelengths at different temperatures can be realized from the photographs of the lantern recorded at different temperatures as shown in Fig. 2e. This change is found to be irreversible, as lowering of the temperature to a normal one does not reset the peak position.

It has been reported that the emission peaks prominently shifted towards red for males of the species *L. praeusta* at 42 ºC^7^, and the winter species *Diaphanes* sp. at 28 ºC^11^, with the conclusion that these changes imply denaturations of the firefly enzymes at or above these temperature optimums. In the case of the presently studied species, as the peak-shift becomes distinct from 34 ºC onwards, we could say that denaturation of the luciferase probably occurs at this temperature. However, at the maximum tolerable temperature of 45 ºC, the change in the intercellular pH might be significant, adding to the red-shift of the spectra. A few reports have put forward propositions on the mechanism of the color change in the firefly luciferase in vitro at varying pH and temperature. It has been suggested that conformational changes of the firefly luciferase at different pHs could be the reason for producing red bioluminescence and changes of kinetics^12,13^. In another report, rigidity of the pH-sensitive luciferase has been proposed to be the reason behind different emission colors^14^. Partial denaturation by heat and low pH has been cited as the probable reason for the redshift in the bioluminescence spectra^15^. Bioluminescence of the firefly *Photinus pyralis* has been shown to change its color at pH 6.8 from yellow-green at 15 ºC to yellow at 25 ºC and then to orange at 34 ºC, which in conjunction with sharp lengthening of the decay time pointed towards denaturation of the luciferase^16^.

At temperatures below 28 °C, values of the peak and FWHM of the spectra remain the same up to approximately 10.5 °C (Fig. 2c,d). Around this temperature, the peak of the spectrum starts shifting towards the shorter wavelength side, with the FWHM becoming narrower. As the temperature is lowered to the minimum of 6.5 °C, at which specimens emit continuous faint light, the peak gets shifted to 553 nm with a minimum FWHM value of 57 nm (Supplementary Table 1). The maximum peak shift towards the lower wavelength side, therefore, is 5 nm. Below 6.5 °C, no light emission from the specimens is observed. Changes towards the side of the lower wavelength could even be noticed easily from the images of the lantern shown in Fig. 2e. Therefore, this figure is apparently a photographic evidence of the change in the emitted wavelength peak due to the change in the temperature to very low and very high values for this species. A general observation is that the emission intensity increases as the temperature is decreased from 28 °C; the intensity reaches a maximum value at approximately 10.5 °C, and thereafter decreases continuously up to 6.5 °C. The change in the peak-value and attainment of the maximum intensity imply that the enzyme luciferase of *S. substriata* possibly gets denatured at about 10.5 °C due to the cooling effect. The changes observed, however, are reversible; when the temperature is increased, the peak goes back to its normal position and intensity.

The change in the emission peak towards red or blue with the change in temperature implies that the relative probabilities of transition from an upper level to the lower levels get affected, and the maximum probability at a high or a low temperature is no longer associated with the usual two levels giving rise to the 558 nm peak at 28 °C. We may now consider temperatures above 34 ºC as high and below 10.5 ºC as low for this species of firefly.

It could be noticed in Fig. 2a,b that the luminescence intensity decreases as the temperature rises up to 34 ºC, though the wavelength peak (558 nm) and FWHM (61 nm) remain unchanged in this range. A decrease in intensity with an increase in temperature has also been noticed in cases of the other three Indian species of fireflies^7, 11,17^. With the increase in temperature, the rate of the biochemical reaction inside the firefly increases and the excitation-emission process becomes more frequent. Since the rate of the firefly chemiluminescence reaction increases, the flash duration decreases which implies a decrease in the lifetime of the upper level of the excited state oxyluciferin^6^. As the steady-state luminescence intensity is proportional to lifetime^18^, the emission intensity decreases. Another possibility is that above 34 ºC, because of the denaturation of the enzyme, only a small number of luciferin molecules take part in the luciferase-luciferin reaction which results in the weak emission-intensity.

**Figure 3:**
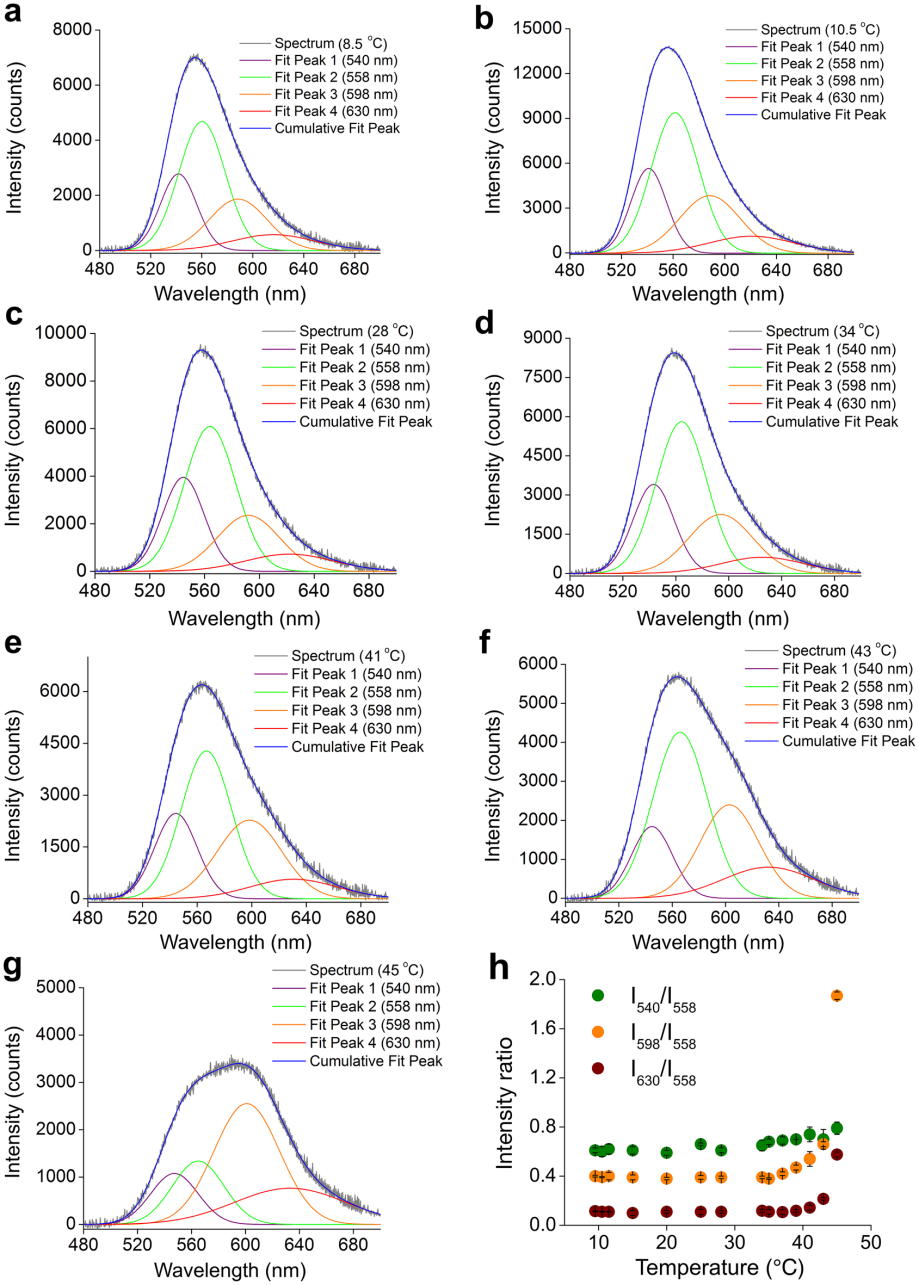
Gaussian fits peaking at 540 nm, 558 nm, 598 nm and 630 nm along with the experimental spectra. (**a**–**g**) Fitted spectra at various high temperatures. At 8.5, 10.5, 28, 34, 41 and 43 ºC, the 558 nm green component is more intense than the other three components. At 45 ºC, the orange component is the most intense one. (**h**) Variation of the measured intensity ratio I_540_/I_558_ (mean ± SD)_,_ I_598_/I_558_ (mean ± SD) and I_630_/I_558_ (mean ± SD) of the Gaussian components with temperature. The intensity ratios I_598_/I_558_ and I_630_/I_558_ increase exponentially as the temperature is increased from 28 ºC to the maximum of 45 ºC, while the change in I_540_/I_558_ is linear. At low temperatures, the spectra do not show any significant change in the ratios, except in I_540_/I_558_.

Variations in bioluminescence peak energies of this firefly over the temperature range of 6.5–45 ºC are presented in Fig. 2f. At the normal laboratory temperature of 28 ºC, the emission shows the bioluminescence peak energy at 2.2244 eV which remains the same up to 34 °C (Supplementary Table 2, Fig. 2b,f). Above this temperature, the energy decreases slowly up to 41 °C, and thereafter rapidly especially from 43 °C onwards, attaining a minimum value of 2.079 eV (598 nm) at 45 °C. At low temperatures, the peak energy does not change up to approximately 10.5 °C; thereafter it increases and attains the maximum value of about 2.2445 eV at 6.5 °C (Supplementary Table 2, Fig. 2d,f). It could be observed from the wavelength-spectra that the spectral shape changes with an increase in temperature above 34 °C (Fig. 2a,b), which becomes clear from 41 °C. The emission intensity decreases at high temperatures, and at 45 °C the spectrum nearly decomposes into two peaks. To analyze the changes with temperatures, we employ the technique of Gaussian curve fitting by assuming four peaks: two green components of 540 nm (2.2986 eV) and 558 nm (2.2244 eV), an orange component of 598 nm (2.0756 eV), and a red component of 630 nm (1.9702 eV). The Gaussian fitted spectra reproduce the experimental spectrum, which explain changes in the spectral shape very well. The Gaussian fitted curves along with the experimental spectra recorded at 8.5, 10.5, 28, 34, 41, 43 and 45 °C are presented in Fig. 3a–g, and the variation of the intensity ratio of the Gaussian components are presented in Fig. 3h with the values given in Supplementary Table 3. The intensity ratios I_598_/I_558_ and I_630_/I_558_ remain approximately equal from 25 to 34 °C, and increase exponentially thereafter with further increase in temperature up to 45 ºC. On the other hand, the intensity ratio I_540_/I_558_ changes linearly with a change in temperature. The gradual fall in the intensity of the green component of 558 nm and the rise in the orange- and red-intensity with the increase in temperature are clearly visible in the Gaussian fitted spectra (Fig. 3a–g). Hence it may be inferred that changes in spectral shapes in the emission spectra at high temperatures are due to the rise in the intensity of the orange and red components, respectively. A study in vitro on bioluminescence emissions of the firefly *Photinus pyralis* has revealed that the intensity of the green component of energy 2.2 eV is the only temperature-sensitive component while the red and orange ones of energies 1.9 eV and 2.0 eV are robust^16^. In the present in vivo study, it is quite clear that the intensities of the orange and red regions are the temperature-dependent components above 34 °C. The peak of each Gaussian component is shifted towards red at high temperatures (Supplementary Fig. 2a) and towards blue at low temperatures, and the FWHM spread of each component increases slightly with an increase in temperature (Supplementary Fig. 2b), similar to the observation of the change in the bioluminescence color due to a change in the pH^19^. It is worth mentioning here that in the temperature-range of 15–34 °C at pH 6.2, 7.0, and 8.0, luminescence energies and widths for green, orange and red components in the Gaussian fit spectra of in vitro bioluminescence of *P. pyralis* do not show any significant temperature dependence. At temperatures below 28 °C in the present case, no significant change in the ratio I_598_/I_558_ and I_630_/I_558_ (Fig. 3h) is observed, which implies that the orange and red components are insensitive to the low temperature.

### Time-resolved measurements at different temperatures

**Figure 4:**
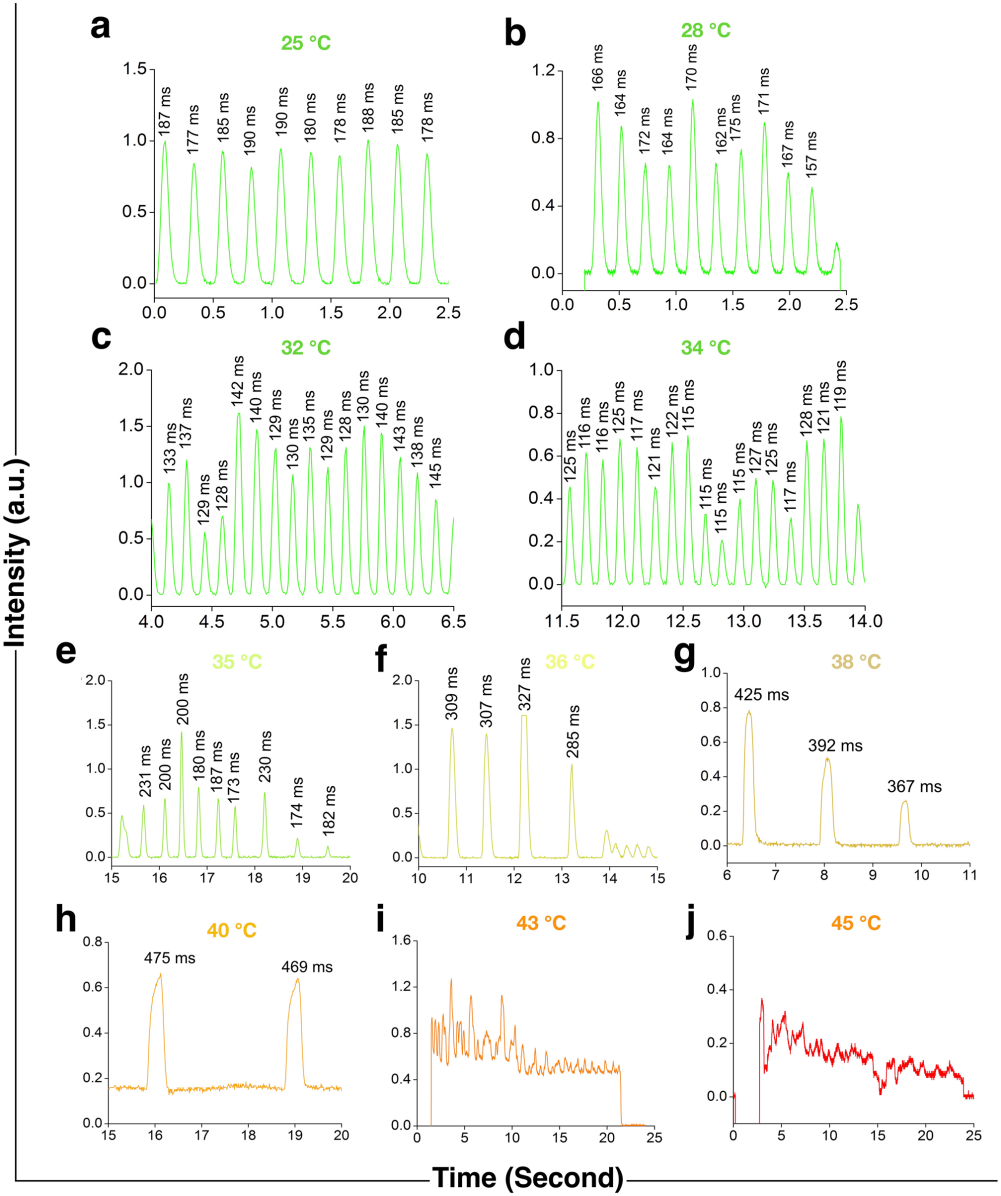
Flashes from the specimens of *S. substriata* at normal and high temperatures. (**a**–**d**) At 25, 28, 32, and 34 °C, the flash duration and the flash interval could be observed to decrease as the temperature increases. Regularity in the interval is sustained. (**e**–**h**) At 35, 36, 38 and 40 °C, the flash duration increases considerably with temperature which probably imply high temperature denaturation of the enzyme luciferase. The flash duration is somewhat uniform. Distortions slowly begin to manifest in the flashes from 38 °C upwards. The duration of a flash is indicated near its peak in this figure, (**a**–**h**). (**i**,**j**) At 43 and 45 °C, pulses are no longer single; these become combination ones and irregular.

Flashes emitted by this firefly at normal and high temperatures are presented in Fig. 4, and the data given in supplementary data 1. Unlike males of *L. praeusta* which usually emit three flashes in a particular sequence, this species emits a number of consecutive flashes in a regular interval of time producing a long pulse train at normal temperatures (Fig. 4a–d, Supplementary Video 1). Flashes from a male *S. substriata* at a certain temperature are found to be considerably broader than those from a male *L. praeusta* at that temperature. For example, in the normal flashing range, its durations of 187 and 147 ms (Supplementary Table 4) are distinctly longer than *L. praeusta*’s 112 and 97 ms at 25 and 30 °C, respectively^6^. Longer flash durations of this species indicate that the chemiluminescence reaction lasts longer, probably proceeds slower, than in *L. praesuta.* Though males of these two species are of the same size, approximately, the lantern-size of *S. substriata* is smaller due to the existence of a small gap between the upper and lower segments, which does not exist in *L. praeusta.* This gap implies the presence of a lower concentration of the luciferin-luciferase combination in the lantern of the presently studied species*.* Therefore, we propose that presence of the lower concentration of this combination in *S. substriata*, compared to *L. praeusta*, probably makes the speed of the reaction slower. It is observed that the flashes at 40 °C are simple, not compound or combination ones, but those appear above the zero level of the oscilloscope. From 40 ºC upwards, no single or ‘clean’ pulses are observed; the ones obtained are noisy and of irregular shapes. The minimum intensity of the flashes remains above the zero level, sometimes lying very close to the zero level, resembling the flashes from the winter firefly *Diaphanes* sp. at its normal flashing temperatures^11^ and from *L. praeusta* at low temperatures^8^. A close observation on the two light emitting segments reveal that the lower segment glows continuously, while only the upper one blinks at these high temperatures. This should be the reason for the intensity never really coming down to the zero level during the ‘off’ time above 40 °C. Effects of low temperature on the flashes of *Diaphanes* sp. and *L. praeusta* have been suggested to be due to the non-uniform proceeding of the neural activity releasing the nerve pulse octopamine, which eventually affect the regular time-resolved flashes. In the present experiment also, the neural activity clearly gets affected at high temperatures. It is well known that after denaturation the number of reactants in a reaction decreases with an increase in temperature, resulting in the excitation of less number of molecules to the upper level. In this case when the temperature goes past 40 °C, the number of luciferin molecules reaching the excited level is probably much less, and because of this the low intensity time-resolved emission profile has been obtained with a noisy appearance. A similar observation has been made at 37–38 ºC for the winter firefly *Diaphanes* sp.^11^.

**Figure 5:**
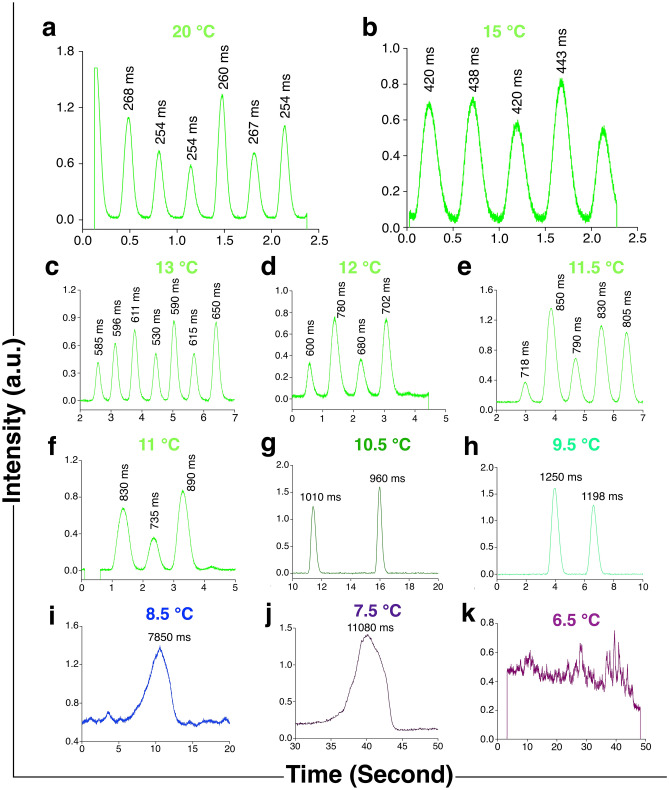
Time-resolved pulses of firefly *S. substriata* at low temperatures. (**a**–**e**) At 20, 15, 13, 12 and 11.5 ºC, the flash-duration, indicated near the peak, increases with the decrease in the temperature from the initial 264 ± 9 ms. The increase is noticeable even to the naked eyes. The flash-interval also clearly increases with decrease in temperature, and regularity in the interval is sustained up to the temperature of 11.5 ºC. (**f**–**h**) At 11, 10.5 and 9.5 ºC, the flashes, though considerably broadened, are still simple, not compound; these are distortion-less. The flash interval is still somewhat regular. (**i**,**j**) At 8.5 and 7.5 ºC, the flashes are distorted. After the appearance of one very broad flash, the second one is not observed. (**k**) At 6.5 ºC, only a continuous noisy output—no flash—is obtained.

Flashes produced at low temperatures are presented in Fig. 5, and the data given in supplementary data 2. It is easily noticeable that when the temperature is lowered from 20 °C, the duration of a single flash increases slowly, and the increment becomes sharp from the temperature of approximately 10.5 °C. At the temperature of 9 °C, the flashes become considerably broad, and in most of those the start- and end-points are not sharp or clear. Some of the specimens stop flashing at this temperature. At temperatures lower than this, the number of flashing specimens become even smaller and their flashes present distorted shapes. When the temperature is further decreased from approximately 8.5 °C, the specimens emit a giant flash, and thereafter emit glows or continuous light of weak intensity. At 7.5 °C, eight such flashes from eight specimens could be recorded; below this temperature, no ‘clean’ single flash is observed. At 6.5 °C, all the insects stop flashing, and only glows could be noticed in their light organs. This weak continuous emission means that the respiration of mitochondria, which densely pack the peripheral cytoplasm of photocytes^20^, is inhibited, and thereby the biochemical trigger oxygen is supplied continuously to luciferin-containing organelles (peroxisomes).

**Figure 6:**
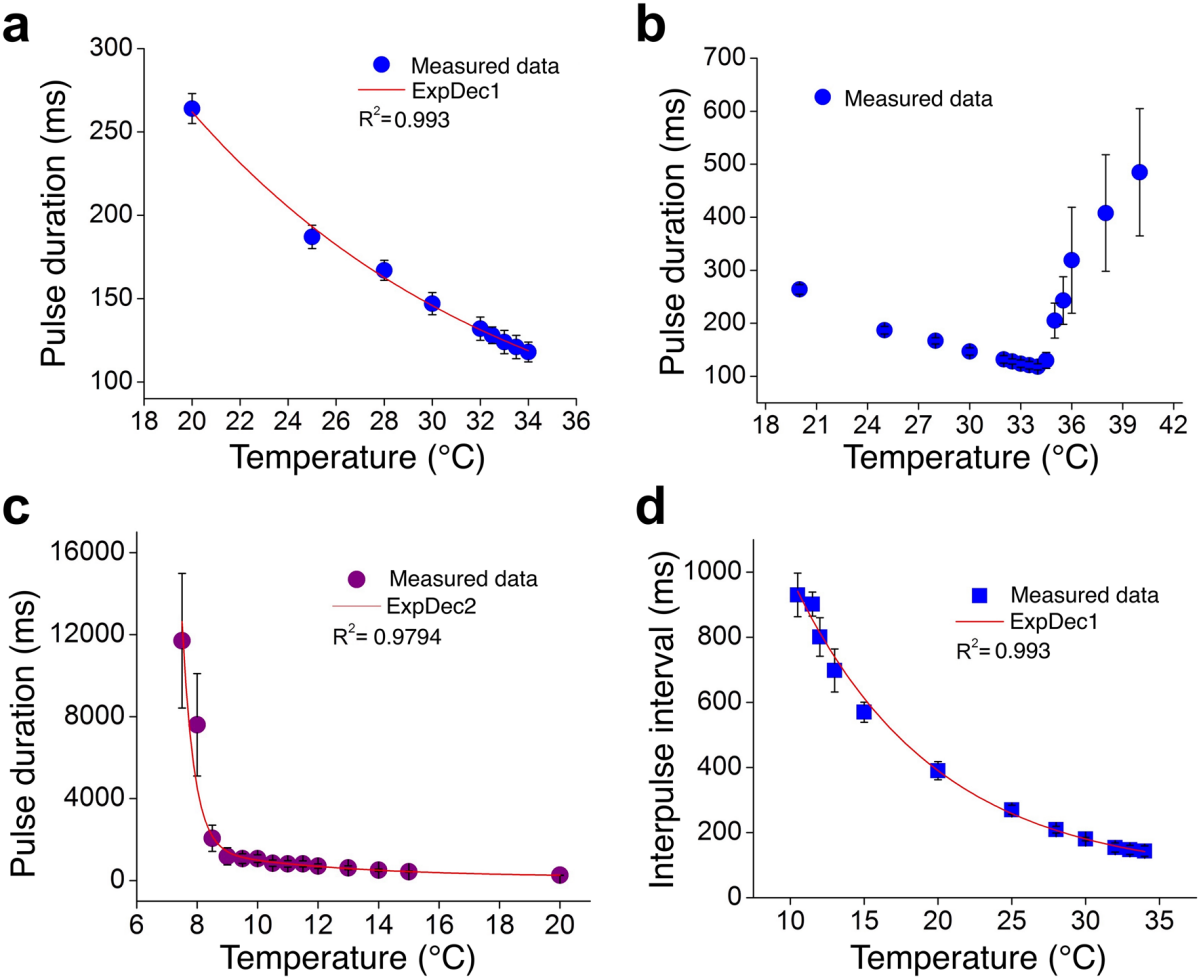
Measurements of time-resolved emissions. (**a**) Variation of the measured flash duration (mean ± S.D.) in the temperature range 20–34 ºC. In this range of temperature, the flash duration decreases exponentially as the temperature increases. This implies that the speed of the luciferase-luciferin reaction increases exponentially up to the temperature optimum shown in (**b**). (**b**) Measured flash duration (mean ± S.D.) at various temperatures in the range of 20–40 ºC. The flash duration is minimum at 34 ºC, which implies the maximum speed of the reaction. Thereafter, the duration increases sharply, indicating sharp decrease in the reaction-speed, with the increase in the temperature. (**c**) Variation of the measured flash duration (mean ± S.D.) in the range of 20–6.5 ºC. The flash duration increases exponentially with the decrease in the temperature up to 7.5 ºC. (**d**) Variation of the inter-flash interval (mean ± S.D.) with temperature from 10.5 to 34 ºC. The variation is exponential.

Variations in the measured pulse duration with temperature over the range of 20–40 ºC are shown in Fig. 6a. In the range of 20–34 °C, changes in the pulse duration are found to be exponential. As already mentioned, over the temperature range of 20–40 °C, the change in the single pulse duration with temperature for the species *L. praeusta* was observed to be linear for its males, indicating that the rate of luciferin-luciferase reaction inside the firefly changed linearly with temperature^6^, and exponential for females suggesting exponential increase in the reaction-rate with temperature^7^. The variation of the decay time of the in vitro bioluminescence of *P. pyralis* in the temperature range of 15–34 °C at pH 7.0 and 8.0 has been reported to be exponential^16^. It is well established that the rate of the enzyme-catalyzed reaction increases exponentially with a simultaneous decrease in the active enzyme through thermal irreversible inactivation^21–23^. It is evident that the flash duration decreases continuously with an increase in temperature up to the temperature optimum of 34 °C, and thereafter increases for further slight increase in temperature (Figs. 4,6b). Recently, minimum flash durations for male and female fireflies of the species *L. praeusta* have been found at 42 °C and 41.5 °C, respectively, above which the durations increase noticeably with slight increases in temperature. Hence 42 °C and 41.5 °C have been considered as the optimum temperatures for male and female fireflies of *L. praeusta*^7^. In the present case, the flash duration becomes minimum, which implies the maximum rate of the luciferase-luciferin reaction, at 34 (± 0.5) ºC. This consolidates the finding that this temperature is the optimum one for the presently studied species of the male firefly. This result is in good agreement with the steady-state emission spectrum, and the change in the flash duration is also found to be irreversible, like the change in the peak position. For the North American species *P. pyralis*, as mentioned already, probable denaturation or deactivation of the enzyme luciferase has been reported above 30 ºC at pH 8.0 as the decay time of the in vitro luciferase-luciferin reaction increases sharply^16^.

The variation in the measured single pulse duration from 20 to 7.5 °C is presented in Fig. 6c. It could be easily observed that the flash-duration increases with a decrease in temperature. It has already been hypothesized that low temperatures slow down the speed of the enzyme-catalyzed bioluminescence reaction, which make the duration longer^6^. In the range of temperature presented in the figure, the increase is exponential. A general observation is that flashes appear in a regular flash-interval, and the interval increases with a decrease in the temperature (Figs. 5,6d); but from 10.5 °C, and more prominently from 9 °C, irregularities occur in the flash-interval. Additionally, changes in the pulse duration from 11.5 to 10.5 °C are very small compared to the ones recorded at other temperatures. Therefore, we could roughly say that 10.5 °C is the temperature up to which the stability of this firefly luciferase is sustained, and is lost at temperatures lower than this. This inference is supported by the values of steady-state intensities of emission (Fig. 2c) and standard deviations of the flashes (Supplementary Table 5). The changes become marked at 9 °C. It has been reported that an enzyme becomes more unstable beyond the transition temperatures of the two states (fold/unfold) due to both cooling and heating effects^24^. Therefore, we speculate that 10.5–9 °C is the range of temperature where denaturation of the enzyme luciferase takes place for this species of firefly. However, it has to be mentioned here that there also exists the possibility of compartmentalization of the active site which gives rise to the blue-shifted spectrum. As in the case of the steady-state emission, changes in the pulse emission are also reversible: the characteristic features come back on raising the temperature above this range.

We realize that the optimum temperature at which thermal effects become prominent could be determined precisely, as the flash duration becomes minimum at this temperature and then increases sharply for small increases from this. Steady-state spectra, showing redshift at this temperature, consolidates this. On the other hand, because of the fact that the flash duration along with the standard deviation go on increasing with lowering of the temperature, exact determination of the optimum temperature producing marked changes in the emitted flashes is difficult. In the present case at 10.5 ºC, steady-state spectra manifest a blue-shift which could possibly be an indication of cold denaturation. But at this temperature, the increase in the standard deviation is not strikingly high and the pulse broadening is just a little bit more marked in the flashes obtained. Hence making a statement that probable protein unfolding starts exactly at this temperature is difficult. It is at 9 ºC that the duration and its fluctuation, that is, standard deviation, increase sharply which should be a confirmation of the instability of the enzyme. Therefore, instead of precisely pinpointing a temperature, it would be safe to indicate a small range of temperature—between 10.5 and 9 ºC.

Average values of inter-flash intervals of the firefly *S. substriata* at temperatures from 10.5 to 34 ºC are shown in Fig. 6d. At the normal laboratory temperature of 28 ºC, the flash interval is measured as 209 ms (Supplementary Table 6); the interval changes exponentially in the range of 20–34 °C. It is easily noticed that the flashes come closer to each other when the temperature rises (Fig. 4), and go away from each other as the temperature decreases from 28 ºC (Fig. 5). This clearly points to the fact that the inter flash interval decreases when the temperature increases, and vice-versa. Above 34 ºC, the interval no longer remains regular—some of the flashes are very closely spaced while some are very widely spaced—thus making a statistical analysis pointless. Similarly, below 10.5 °C, flash intervals vary so much that their measurements have to be discarded at those temperatures. In a communication, it has been shown that the average normal flash interval of the firefly decreases with an increase in temperature^25^. In the present case, we propose that a change in temperature over the range of 9–34 °C affects the rate of regular flow of oxygen to the light producing organelles, and this changes the inter flash interval. Cold denaturation at low temperatures and heat denaturation at high temperatures are the probable reasons for the irregularities in flash intervals from below 10.5 °C and above 34 °C.

Few proteins have been reported to undergo cold denaturation above the freezing point of water in the absence of denaturants. The yeast frataxin homologue 1 (Yfh1), the C-terminal domain of ribosomal protein L9, the scaffold protein for iron-sulfur (Fe-S) cluster biosynthesis in *Escherichia coli*, IscU, and the human immunodeficiency virus-1 (HIV-1) protease are the ones found to exhibit this^26–29^. We propose for addition of the firefly luciferase in the body of a live firefly to this list. Extraction of luciferase from the light organs of dead fireflies and determination of the relevant thermodynamic parameters at these temperatures would confirm this proposition; but that would be a study in vitro, the real flashing situation no longer existing.

### Choices of appearance-time and habitat

The red shifting temperature of 34 °C for *S. substriata* comes in between the ones for *L. praeusta* (42 ºC) and *Diaphanes* sp. (28 ºC). These temperatures give the reason why *L. praeusta* fireflies could be active in the hot summer season just after sunset when the ambient temperature remains high, while *Diaphanes* sp. fireflies could be active in the cold winter seasons only. As the species *S. substriata* becomes active usually 1 to 2 h after sunset, there is enough time for the ambient temperature to cool down to a few degrees below the temperature optimum. In a study on 55 species of North American (temperate zone) fireflies, it has been found that ‘dark-active’ fireflies emit green bioluminescence and ‘dusk-active’ species emit yellow, in general^30^. The two broad groups of species, ‘early-starting’ (dusk-active) and ‘late-starting’ (dark-active), have been defined as the one that begins the flashing activity in advance of 30 min after sunset and the one that begins after this, respectively. Of 32 dark-active species observed in that study, 23 have been found to emit green light (λ_max_ ≤ 558 nm) and 21 of 23 dusk-active species emit yellow light (λ_max_ ≥ 560 nm). The species *S. substriata*, barely falling in the first category, is generally observed to be dark-active. However, on the few cloudy or rainy summer days when the temperature at the time of sunset becomes 30 ºC or less, one or two specimens of it have been sighted just about half an hour after the sunset. That is, it becomes almost dusk-active, and there is little to differentiate the appearance times between this species and the dusk-active *L. praeusta* inhabiting the same locality. Generally, though, the majority of *L. praeusta* fireflies come out before sunset on *any* day, while the majority of the *S. substriata* fireflies comes out roughly 45 min after sunset in *those* days. Hence, we propose that the peak-wavelength, though generally determining a firefly’s active time, does not determine its time of coming out—the temperature indicating denaturation of the luciferase is the chief deciding factor. This temperature also gives the reason for their choice of the habitat. In this locality, the temperature of the hottest days during the summer goes past the optimum denaturation-temperature of 34 ºC. Shades from branches and leaves of the trees in the banks of the big ponds help to keep the temperature within the temperature optimum of this firefly. In the hot days, these fireflies have been observed to start flashing 2–3 h, sometimes more than 3 h, after sunset, sitting on leaves or branches of trees. We hypothesize that different enzyme-structures of different species give rise to different temperatures of denaturation, along with different wavelength-peaks, and these are the reasons of their choice of different habitats and times of activeness.

As this species finds temperatures above 30 °C difficult, and above 34 °C impossible, to bear, a question immediately arises: Why, then, it does not choose a place where the temperature is cooler than this? The answer to this question probably lies in the cold denaturation of the enzyme luciferase. Since this is indicated between 10.5 and 9 °C, the insect obviously has to maintain some margin. From observations of 3 years, we conclude that the normal flashing-range of temperature for this species is 25–30 °C. It could be noticed that the change in the pulse duration is approximately linear from high temperature optimum of 34 °C to 25 °C, and the linearity is lost below 25 °C (Fig. 4b, Supplementary Table 4). We speculate that the loss of linearity—which implies longer lasting or probably slower proceeding reaction—forces the firefly to stop flashing at temperatures lower than this. The other species *L. praeusta*, which exists in this locality in plenty, is also no longer seen at around the same time at the onset of the winter. The linearity in the duration-change in flashes for males of that species has also been found to be lost from approximately the same temperature^7^. Determining the blue-shifting temperature for that species, or for that matter for all the species of the world, will clarify how much margin a summer species of firefly normally keeps before vanishing from our sights for that year. In late October, when this firefly disappears for the season, the minimum temperature in the region goes down to roughly 20 °C. Thus, this species needs temperatures about 10 °C more than the low temperature optimum to be found in the locality, and more than 24 °C after sunset to ‘warm up’ for flashing. As for the high temperature part, *L. praeusta* fireflies come out just after sunset—the temperature at that time in this locality, though normally 32–33 °C, could even be about 35 °C for a day or two in the hottest days. That is, *L. praeusta* fireflies maintain a margin of 7 °C while *S. substriata* fireflies maintain a difference of at least 4 °C from their high temperature optimums. Hence we put forward the general proposition that the high temperature optimum decides the firefly’s active time, and the high and low temperature optimums together decide its choice of the habitat as far as temperature-suitability is concerned.

## Conclusion

When the temperature is increased to 34 °C, the emission spectrum shows a redshift of the wavelength peak and emitted pulses show the minimum width which increases sharply for further small increase in temperature. These probably indicate thermal denaturation of the enzyme luciferase. We propose that this is the reason for the species being dark-active in summers. On the other hand, blue shifting of the peak and considerable broadening with fluctuations of the flashes at 10.5–9 °C point towards possible occurrence of cold denaturation in this range of temperature. These two effects, apart from availability of food, of course, give the reason why this species finds this particular place comfortable to dwell. We have to have the enzyme structures vis-à-vis the emission wavelength peaks and different active times of *all* the species of fireflies for drawing the complete picture.

## Methods

### Sample collection

Before the experiment, a couple of adult male specimens of the firefly *S. Substriata* were captured on the banks of two big ponds lying side by side in the campus of Gauhati University about one and a half hours after the sunset. These insects were observed to fly above water near the bank, and rest on leaves of trees considerably above the ground, and whenever they came flying to a catchable height on the ground of the bank, they were caught. The collected specimens were then brought to the laboratory, approximately 1 km from the location. A strong and intensely flashing one was selected for experiments. This species has the characteristic that it emits light only when in motion, never when immobile! Therefore, arrangements had to be made to make the firefly move for recording spectra in both steady-state and flash emissions.

### Recording of the firefly spectra

#### At the laboratory temperature

For recording emission spectra, an already calibrated high-resolution spectrometer (Ocean Optics HR4000) was used. The selected specimen was put inside a 0.5 ml micro-centrifuge tube. A piece of cotton was inserted in the tube so that the firefly could move in a very small volume. A small hole of size nearly equal to that of the ends of a fiber optic cable (cable number: QP200-2-UV–VIS, EOS-A6282132-2) was made on the conic surface of the tube. When the lantern of the inserted specimen faced the hole, the emitted light entered through the entrance face of the fiber and was collected by it. The Ocean Optics software Spectra suite was used to visualize and record the spectra. The data obtained were saved in ProcSpec files. A temperature sensor of resolution 0.1 ºC, made by using the IC LM35 connected to a digital multimeter (MASTECH MAS 830L), was used to note the temperature. The exact temperature at the location of the firefly was determined by inserting the sensor inside the centrifuge tube and waiting till the temperature became reasonably stable. The spectral analysis was carried out by using Microsoft Origin 8.0. Spectra in the energy scale were plotted by converting each data point in the wavelength spectra to energy using the equation $E=hc/\lambda$.

For recording time profiles of flash emissions, a transparent cylindrical cavity (petri dish, 35 mm) was used. To ensure air flow through the cavity, a number of small holes were made on the flat and curved surfaces of the cavity. A digital storage oscilloscope (Tektronix TDS 2022C) along with a photomultiplier tube (Hamamatsu H10722 with power supply C10709) were used to record the time-resolved spectra. The cavity was attached to the PMT using a sellotape, and the surface with no holes faced the light entering face of the PMT. The specimen was allowed to move freely inside the cavity, and whenever it came to the location of the light entering face of the PMT, flashes were observed and recorded in the DSO; those were saved in a USB device (HP v215b, 16 GB) as .CSV extension files. As the light intensity from this insect changed with time, the control voltage applied to the PMT was changed from 200 to 400 V to have good flash-amplitudes. The same digital thermometer, kept attached to the cavity, was used to look at the temperature. Before recording the flashes, a calibration of the inside and outside temperatures of the cavity was carried out which showed a difference of 1 ºC—the inside one being more during the lowering and less during the raising of temperature. The inside temperature, of course, was considered for the experiment. Time-resolved measurements, as in the case of the steady-state ones, were carried out with the help of Microsoft Origin 8.0.

#### At high temperatures

For producing high temperatures, a 1–2 kw fan heater (Orpat OEH-1260) was used. By varying the distance between the firefly and the heater, different temperatures were realized. The centrifuge tube was kept at a fixed position, and the heater was moved towards it to increase the temperature. After noting down the temperature inside the tube, the position of the heater was marked, and the sensor removed and attached to outside of the tube using a sellotape. At each distance from the heater (Supplementary Table 7), the fluctuation in the temperature was found to be ± 0.2 ºC. The insect was acclimatized for about 10 min in each stepwise increase of temperature. The maximum temperature of 45 ºC used in the experiment was obtained at the distance of 52 cm from the location of the firefly. At this temperature, most of the specimens were found in the dying stage, and a few died; the specimens displayed continuous glows in their light organs—not flashes—for a couple of seconds to a maximum of 5 min.

#### At low temperatures

For experiments at low temperatures, a specimen along with the same micro-centrifuge arrangement was brought near the window of the air conditioner (ORPAT). The specimen was acclimatized for about ten minutes at a certain low temperature, that is, at a certain distance from the window of the AC. The distance between the insect and the AC was decreased in steps to decrease the temperature at the location of the firefly (Supplementary Table 8). The minimum temperature obtained was 12 ± 1 ºC with the fluctuation of ± 0.2. To decrease the temperature below 12 ºC, a cardboard of surface area 26.5 cm × 15 cm covered with an aluminium foil was made to incline with the horizontal surface (Supplementary Fig. 1). The cool air coming from the AC got reflected in the cardboard and moved towards the micro-centrifuge arrangement of the sample. This reflected air began to lower the temperature at the location of the firefly, and a minimum value of 9 ± 0.5 ºC could be obtained. To lower the temperature further, one side of the reflector was closed with another cardboard covered with an aluminium foil of the same area, so that the diverging cool air coming from the AC got reflected at this second reflector and thereby lower the temperature to a minimum value of 8 ± 0.5 ºC. The temperature was lowered even further by closing the other side with another reflector. The reflectors on either side of the first reflector were held fixed by mechanical supports such that they do not fall due to the pressure from air coming from the AC. The three reflectors and the horizontal surface along with the window of the AC form a three dimensional air cooled chamber, similar to a triangle-shaped prism. Hence most of the cooled air got reflected and circulated inside the chamber and the temperature dropped down to the minimum value of 6.5 °C. No further decrease in the temperature was possible. This air-cooling chamber arrangement to lower the temperature below 12 ºC might appear to be not so robust, but it was quite effective, as the effect of temperature on the light emission from this species, just like from *L. praeusta*^6^, was observed to be instantaneous. Spectra were recorded at each 0.5 ºC fall in the temperature. From approximately 8.5 ºC, spectra were recorded for each flash as after producing one flash by the fireflies, their light organs produced continuous glows only.

Different sets, 30 in each, of specimens were used in the steady-state and time-resolved experiments. After the experiments, the specimens were set free.

### Curve fitting

To analyze the spectral changes, we carried out Gaussian curve fitting in Origin 8.0. We assumed four peaks around 540 nm, 558 nm, 598 nm and 630 nm to reproduce the experimental spectrum. Three parameters, viz., peak, FWHM and area of each Gaussian component, were adjusted so that their summation spectrum coincided with the experimental spectrum. Most of the fittings gave correlation coefficients above 0.999, except the few obtained in the range of 0.991–0.998. The lower correlation coefficients were obtained due to the relatively low S/N ratio of the spectra, which were recorded at relatively high and low temperatures.

## Supplementary Information

Supplementary Information 1.

Supplementary Information 2.

Supplementary Information 3.

Supplementary Video S1.

## Data Availability

Data supporting the Fig. 3b–e are given in the Supplementary Information. Rest of the data supporting the findings of this study are with the first named author, and could be made available upon request.

## References

[1] Goswami A, Phukan P, Gohain Barua A (2019). Manifestation of peaks in a live firefly flash. J. Fluoresc..

[2] Gohain Barua A, Rajbongshi S (2010). The light of the firefly under the influence of ethyl acetate. J. Biosci..

[3] Saikia D, Bora MP (2020). Nonlinear model of the firefly flash. Nonlinear Dyn..

[4] Lloyd JE (1973). Fireflies of Melanesia: Bioluminescence, mating behaviour and synchronous flashing (Coleoptera: Lampyridae). Environ. Entomol..

[5] Iguchi Y (2010). Temperature-dependent geographic variation in the flashes of the firefly *Luciola cruciata* (Coleoptera : Lampyridae). J. Nat. Hist..

[6] Sharma U, Goswami A, Phukan M, Rajbongshi SC, Gohain Barua A (2014). Temperature dependence of the flash duration of the firefly *Luciola praeusta*. Photochem. Photobiol. Sci..

[7] Rabha MM, Sharma U, Goswami A, Gohain Barua A (2017). Bioluminescence emissions of female fireflies of the species *Luciola praeusta*. J. Photochem. Photobiol. B: Biol..

[8] Sharma U, Goswami A, Rabha MM, Gohain Barua A (2016). In vivo bioluminescence emissions of the firefly *Luciola praeusta* at low temperatures. J. Photochem. Photobiol. B: Biol..

[9] Gohain Barua A, Iwasaka M, Miyashita Y, Kurita S, Owada N (2012). Firefly flashing under strong static magnetic field. Photochem. Photobiol. Sci..

[10] Seliger HH, Buck JB, Fastie WG, McElroy WD (1964). The spectral distribution of firefly light. J. Gen. Physiol..

[11] Rabha MM, Sharma U, Gohain Barua A (2020). Bioluminescence emissions from the Indian winter species of firefly *Diaphanes* sp. J. Biosci..

[12] Viviani VR (2008). The structural origin and biological function of pH-sensitivity in firefly luciferases. Photochem. Photobiol. Sci..

[13] Nakatsu T, Ichiyama S, Hiratake J, Saldanha A, Kobashi N, Sakata K, Kato H (2006). Structural basis for the spectral difference in luciferase bioluminescence. Nature.

[14] Oliveira G, Viviani VR (2019). Temperature effect on the bioluminescence spectra of firefly luciferases: Potential applicability for ratiometric biosensing of temperature and pH. Photochem. Photobiol. Sci..

[15] McCapra F, Gilfoyle DJ, Young DW, Church NJ, Spencer P, Campbell AK, Kricka LJ, Stanley PE (1994). The chemical origin of colour differences in beetle bioluminescence. Bioluminescence and Chemiluminescence: Fundamentals and Applied Aspects.

[16] Mochizuki T, Wang Y, Hiyama M, Akiyama H (2014). Robust red-emission spectra and yield in firefly bioluminescence against temperature changes. Appl. Phys. Lett..

[17] Goswami A, Sharma U, Rabha MM, Rajbongshi SC, Gohain Barua A (2015). Steady-state and time-resolved bioluminescence of the firefly *Asymmetricata circumdata* (Motschulsky). Curr. Sci..

[18] Lackowicz JR (2004). Principles of Fluorescence Spectroscopy.

[19] Ando Y (2007). Firefly bioluminescence quantum yield and colour change by pH-sensitive green emission. Nat. Photonics.

[20] Trimmer BA (2001). Nitric oxide and the control of firefly flashing. Science.

[21] Copeland RA (2000). Enzymes: A Practical Introduction to Structures, Mechanism, and Data Analysis.

[22] Dixon M, Webb EC (1979). Enzymes.

[23] Garrett RH, Grisham CM (2010). Biochemistry.

[24] Noelting B (2005). Protein Folding Kinetics: Biophysical Methods.

[25] Charles D, Snyder AVTH (1920). The flashing intervals of fireflies—its temperature coefficient—an explanation of synchronous flashing. Am. J. Physiol..

[26] Pastore A (2007). Unbiased cold denaturation: Low- and high-temperature unfolding of yeast frataxins under physiological conditions. J. Am. Chem. Soc..

[27] Li Y, Shan B, Raleigh DP (2007). The cold denatured state is compact but expands at low temperatures: Hydrodynamic properties of the cold denatured state of the C-terminal domain of L9. J. Mol. Biol..

[28] Bothe JR (2015). The complex energy landscape of the protein IscU. Biophys. J..

[29] Rösne HI (2017). Cold denaturation of the HIV-1 protease monomer. Biochemistry.

[30] Lall AB, Seliger HH, Biggley WH, Lloyd JE (1980). Ecology of colors of firefly bioluminescence. Science.

