# Supplementary material for: Light from a firefly at temperatures considerably higher and lower than normal
Source: Sci Rep. 2021 Jun 14;11:12498. doi: 10.1038/s41598-021-91839-3 (PMC8203691; doi:10.1038/s41598-021-91839-3)
Supplement: Supplementary file 1 — Supplementary Information 1. [file 41598_2021_91839_MOESM1_ESM.pdf]

Supplementary Materials

Light from a firefly at temperatures considerably higher and lower than normal

Mana Mohan Rabha[1], Upamanyu Sharma[2], Anurup Gohain Barua[2]*

[1]Department of Physics, Pandit Deendayal Upadhyaya Adarsha Mahavidyalaya, Behali, 784184, India
[2]Department of Physics, Gauhati University, Guwahati, 781014, India

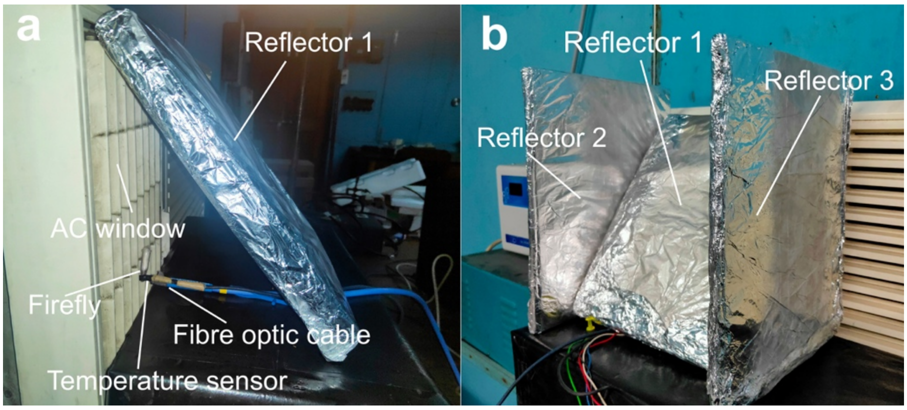

Supplementary Figure 1. Reflectors and the Window of the AC**.** (**a**) Reflector 1 inclined with the horizontal surface. (**b)** Reflector 1, 2 and 3 forming a structure which gives a prism-like shape. Reflector 1 lowers the temperature to 9.5±0.5 ºC, and the Reflector 2 and 3 lower the temperature to 5 ºC. The reflectors are covered with aluminum foil for heat insulation.

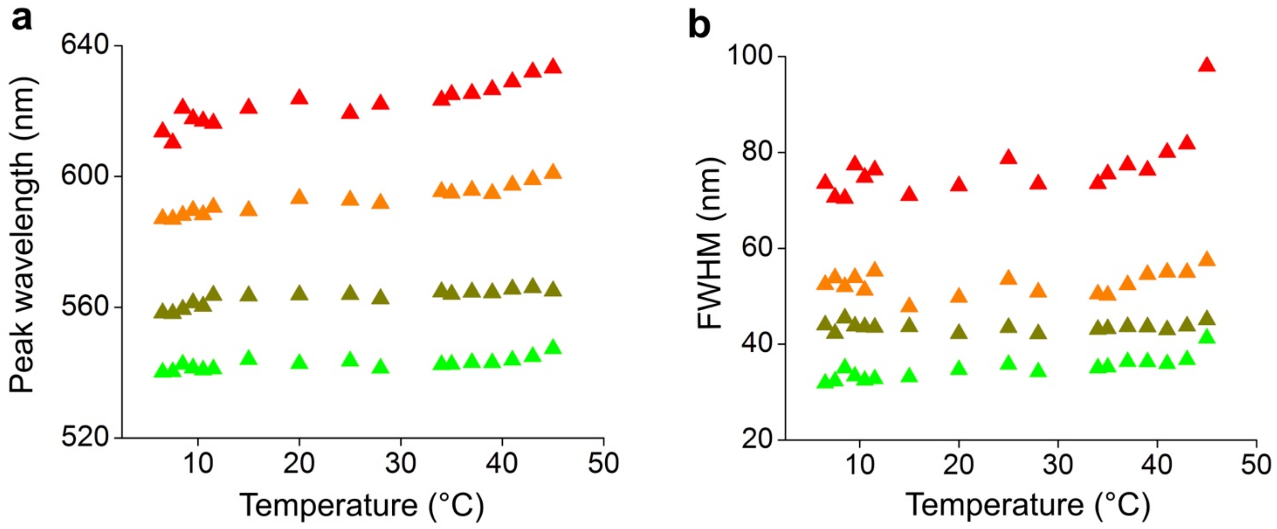

Supplementary Figure 2. Parameters of Gaussian curve fits. (**a**) Peak wavelength of each Gaussian component (▲ :540 nm peak, ▲ : 558 nm peak, ▲ : 598 nm peak and ▲ : 630 nm). (**b)** FWHM of the gaussian components (▲ :540 nm peak, ▲ : 558 nm peak, ▲ : 598 nm peak and ▲ : 630 nm).

Supplementary Table 1. Measured values of peak and FWHM of the steady-state mission spectra at various temperatures.

**Temperature (°C)**	**Peak (nm)**	**FWHM (nm)**	**FWHM range (nm)**
6.5	553±0.3	57±0.4	530±0.1 - 587±0.44
7.5	554.5±0.07	58±0.33	531.5±0.08 - 589.5±0.30
8.5	555.5±0.15	58.5±0.35	531.5±0.19 - 590.5±0.46
9.5	556.5±0.5	59±0.09	531.5±0.24 - 590.5±0.21
10.5	557±0.02	59.5±0.18	532±0.1 - 591.5±0.30
11.5	558±0.06	61±0.041	532.5±0.04 - 593.5±0.04
15	558±0.059	61±0.04	532.5±0.041 - 593.5±0.05
20	558±0.062	61±0.043	532.5±0.05 - 593.5±0.045
25	558±0.065	61±0.04	532.5±0.039 - 593.5±0.03
28	558±0.065	61±0.04	532.5±0.04 - 593.5±0.04
34	558±0.15	62±0.29	532.5±0.30 - 593.5±0.30
35	560.5±0.31	63±0.19	533±0.20 - 596±0.30
37	561.5±0.25	63±0.22	533.5±0.09 - 597±0.27
39	561.5±0.38	65.5±1.15	533.5±0.06 - 598±0.31
41	564±0.30	70±0.30.71	534.5±0.25 - 605±0.30
43	565±0.12	78±0.30.5	535±0.3 - 613±0.29
45	598±0.2	96±0.34	539±0.2 - 635±0.1

.

Supplementary Table 2. Measured peak energies with standard deviations at various temperatures
.

Temperature (℃)	Peak energy (eV)	Standard deviation (eV)
6.5	2.2445	0.15
7.5	2.2385	0.17
8.5	2.2344	0.08
9.5	2.2304	0.024
10.5	2.2228	0.024
11.5	2.2224	0.014
20	2.2224	0.01
25	2.2224	0.012
28	2.2224	0.01
34	2.2224	0.018
35	2.2145	0.04
37	2.2125	0.05
39	2.2105	0.03
41	2.2007	0.04
43	2.1969	0.1
45	2.079	0.18

Supplementary Table 3. Measured Values of I_{540}/I_{558}, I_{598}/I_{558}, and I_{630}/I_{558} at various temperatures.

Temperature (°C)	I_{540}/I_{558}	Standard Deviation	I_{598}/I_{558}	Standard Deviation	I_{630}/I_{558}	Standard Deviation
6.5	0.49	0.04	0.39	0.02	0.11	0.005
7.5	0.52	0.02	0.383	0.02	0.1	0.001
8.5	0.6	0.05	0.385	0.005	0.12	0.01
9.5	0.61	0.02	0.4	0.007	0.115	0.003
10.5	0.6	0.04	0.392	0.0046	0.11	0.0035
11.5	0.62	0.04	0.4	0.03	0.11	0.003
15	0.61	0.03	0.39	0.02	0.1	0.02
20	0.59	0.02	0.38	0.01	0.11	0.01
25	0.67	0.01	0.39	0.015	0.11	0.02
28	0.66	0.02	0.39	0.01	0.11	0.01
34	0.65	0.044	0.39	0.01	0.118	0.01
35	0.68	0.022	0.38	0.006	0.11	0.018
37	0.69	0.03	0.42	0.015	0.107	0.019
39	0.699	0.001	0.47	0.02	0.118	0.01
41	0.74	0.06	0.54	0.06	0.144	0.02
43	0.7	0.08	0.66	0.02	0.215	0.01
45	0.79	0.05	1.869	0.03	0.576	0.02

Supplementary Table 4. Flash duration with standard deviation at various high temperatures.

Temperature (°C)	Flash duration (ms)	Standard deviation (ms)	Number of flashes (*n*)	Number of Specimen
25	187	7	557	30
28	167	6	615	30
30	147	6.7	450	30
32	132	7	245	30
32.5	128	5	222	30
33	124	7	223	30
33.5	121	7	201	30
34	118	6	170	28
34.5	130	15	114	28
35	205	33	97	22
35.5	243	45	55	22
36	319	100	42	14
38	408	110	38	12
40	485	120	16	8

Supplementary Table 5. Flash duration with standard deviation at low temperatures.

**Temperature (°C)**	**Flash duration (ms)**	**Standard deviation (ms)**	**Number of flashes (*n*)**	**Number of Specimen**
20	264	9	437	30
15	437	24	207	30
14	512	55	158	30
13	625	94	126	30
12	712	108	113	30
11.5	815	148	110	30
11	820	155	55	24
10.5	851	150	39	24
10	1072	185	33	18
9.5	1077	238	31	18
9	1185	420	31	12
8.5	2065	641	20	8
8	7600	2500	15	8
7.5	11700	3280	8	8

Supplementary Table 6. Values of average inter-pulse intervals and their standard deviations at different temperatures.

**Temperature (°C)**	**Inter-pulse interval (ms)**	**Standard deviation (ms)**	**Number of pulses (*n*)**
10.5	930	67	40
11.5	902	37	73
12	801	59	83
13	698	66	91
15	570	31	156
20	390	28	221
25	270	14	278
28	209	10	341
30	180	5	293
32	154	13	101
33	147	14	87
34	143	16	72

Supplementary Table 7. Temperatures measured by the sensor IC LM35 placed at various distances from the window of the heater.

**Distance from the heater (cm)**	**Temperature (°C)**
235±1	30±0.2
170±1	33±0.2
151±1	34±0.2
135±1	35±0.2
115±1	37±0.2
101±1	39±0.2
87±1	41±0.2
68±1	43±0.2
52±1	45±0.2

Supplementary Table 8. Temperatures measured by the sensor at various distances from the AC.

**Distances from the window of the AC (cm)**	**Temperature (°C)**
0	12±0.2
18±2	13±0.2
24±2	14±0.2
30±2	15±0.2
40±2	20±0.2
60±2	25±0.2

Supplementary Video 1. Flashes from a specimen of the firefly *Sclerotia substriata* at a normal temperature of 28 °C. Video shows that the specimen emits a number of flashes at a fairly regular interval of time, which are shown graphically in Fig. 4.

Supplementary Data 1. Recorded data of Figure 4. Data for the flashes emitted at 25, 28, 30, 32, 34, 35, 36, 38, 40, 42 and 45 ºC.

Supplementary Data 2. Recorded data of Figure 5. Data for the flashes emitted at 20, 15, 13, 12, 11.5, 11, 10.5, 9.5, 8.5, 7.5 and 6.5 ºC.
